# Personality disorders in individuals with functional seizures: a systematic review

**DOI:** 10.3389/fpsyt.2024.1411189

**Published:** 2024-08-01

**Authors:** Ilaria Sammarra, Iolanda Martino, Laura Marino, Francesco Fortunato, Antonio Gambardella

**Affiliations:** Institute of Neurology, Department of Medical and Surgical Sciences, Magna Graecia University, Catanzaro, Italy

**Keywords:** functional seizures, personality disorders, cluster B personality disorder, borderline personality disorder, emotion dysregulation, dialectical-behavior therapy

## Abstract

Functional seizures (FS) are classified as conversion disorders in the DSM-5 and dissociative disorders in the ICD-11, showing a multifactorial psychopathology with various psychiatric comorbidities, such as depression and anxiety. Several studies have found a correlation between FS and personality disorders, mainly those in cluster B. Within this cluster, borderline personality disorder (BPD) or borderline personality traits are the most prevalent in FS. Emotion dysregulation is a hallmark of BPD and is commonly reported in individuals with FS. Cluster C personality disorders, such as avoidant or obsessive-compulsive disorders, have also been reported in FS. In this review, we aim to evaluate the relationship between FS and personality disorders. Assessing personality disorders in the context of FS is relevant for determining the most appropriate intervention. Cognitive-behavioral therapy (CBT) is considered the first-line approach to treating FS. Among various CBT strategies, dialectical behavior therapy, which specifically targets emotion dysregulation, may be helpful for individuals with BPD. Future research should assess the advantages of systematically evaluating personality disorders in FS to address specific treatment planning and evaluate its effectiveness on seizure recurrence, psychological comorbidities, and quality of life.

**Systematic review registration:**

https://www.crd.york.ac.uk/PROSPEROFILES/509286_STRATEGY_20240203.pdf, identifier CRD42024509286.

## 1 Introduction

Psychogenic nonepileptic seizures, also known as functional seizures (FS), are defined as paroxysmal altered motor, sensory, autonomic, and/or cognitive signs and symptoms that resemble an epileptic seizure but are not caused by ictal epileptiform activity (1). FS are classified as a subgroup of conversion disorders (functional neurological symptom disorder) according to the DSM-5 (2) or as a dissociative neurological symptom disorder with non-epileptic seizures in the ICD-11 (3). The diagnosis reaches the highest certainty level recording a typical event at video-EEG (1). FS are frequently mistaken for epilepsy, delaying the correct diagnosis by an average of 7 years (4, 5). The misdiagnosis leads to inappropriate treatment, with a significant impact on quality of life, morbidity, and healthcare costs (1, 6). In the general population, FS have an estimated incidence of 1.4–4.9/100,000/year and a prevalence between 2 and 33 cases per 100,000 (7). Several theoretical models have been proposed to explain FS development (8, 9). Initially interpreted as a dissociative phenomenon, FS have been derived from a breakdown in psychological integration in response to intense stress or emotion, appearing as a sensorimotor flashback when traumatic dissociated material comes into consciousness (7). Accordingly, the prevalence of traumatic life events varied from 44% to 100% in FS. In particular, sexual abuse is three times more common among FS individuals, ranging from 6% to 85% (7, 10). In addition, childhood emotional neglect, defined as the carelessness of the affectional needs of a child, demonstrated a strong association with FS. Noteworthy, not all individuals reported past exposure to traumatizing events, configuring trauma as neither a necessary nor sufficient condition. Subsequent theory interpreted FS as avoidant/defensive reaction in response to overwhelming situations or traumatic experiences in individuals with low capacity for coping stressful life events (7). A variant of this model interprets FS as a physical component of emotional states not recognized or misinterpreted by individuals due to their inability to identify and/or name emotions (i.e., alexithymia) seen as unacceptable. A further model explains FS as learned behavior maintained by positive or negative reinforcement thanks to intrinsic/extrinsic benefits, like reducing anxiety, relieving duties, or getting attention (8). Recently, “integrative cognitive model” conceptualized FS as behavioral paroxysms resulting from automatic activation of learnt mental representations, defined “seizure scaffold” (7). In detail, an attack’s semiology depends on the content of the scaffold, acquired from internal (as direct symptom experience) or external (as attendance of symptom) sources (11).

FS comprehend concomitant heterogeneous psychiatric disorders, ranging from 53 to 100% (12, 13), and being able to represent predisposing/precipitating factor, underlying etiology, or consequence (14). Depression demonstrated a prevalence rate varying between 8.9% and 100% (13). Suicide risk has been shown to be greater in people with FS compared to the general population (15). Furthermore, the prevalence of anxiety disorders varied between 4.5% and 70%, including panic disorder and generalized anxiety disorder. FS individuals meeting the criteria for post-traumatic stress disorder (PTSD) ranged from 7% to 100% (13). Personality disorders (PDs) demonstrated a high prevalence among people with FS, up to 75% in some reports (13). Within PDs, cluster B, mainly borderline PD (BPD), appears as the most common personality phenotype (15, 16). Interestingly, FS and BPD seem to have some mutually common aspects, sharing a history of traumatic experiences, depression, and PTSD (17). Moreover, emotional dysregulation, considered a BPD hallmark, is commonly described in FS (18). Likewise, cluster C PDs, such as avoidant or obsessive-compulsive, have also been reported in FS (9). Assessment of PDs in the context of FS could be particularly relevant in choosing therapeutic intervention.

As widely recognized, psychotherapy, including cognitive-behavioral therapy (CBT), is the most commonly used approach to treating FS (19). Dialectical-behavior therapy (DBT) is a form of CBT specifically developed for BPD targeting emotion dysregulation and has proved efficacy in FS (9, 20). Therefore, investigating BPD in FS individuals could be relevant to tailoring a therapeutic approach. Nevertheless, few studies evaluated the frequency of PDs in individuals with FS using DSM IV/V criteria. Moreover, systematic analysis of PD clusters found in FS is currently unavailable (13).

This systematic review aims to assess the following in the adult FS population: I) prevalence of PDs diagnosed according to DSM-IV/V or ICD-10/11; II) frequency of clusters A, B, and C in studies evaluating personality phenotypes; and III) PDs and their cluster rates compared to individuals with epilepsy across studies considering both groups.

## 2 Materials and methods

### 2.1 Search strategy and selection criteria

This review, following PRISMA guidelines (21), focuses on primary research articles published between 1950 and 2024. Studies excluded from this review include unpublished research, review articles, editorials, letters, case studies, case reports with less than five individuals, and meta-analyses. The protocol is available online on PROSPERO (https://www.crd.york.ac.uk/PROSPEROFILES/509286_STRATEGY_20240203.pdf) with registration number: CRD42024509286.

Databases PubMed, OVID Medline, and PsycINFO were searched, including the following terms:

(“PNES” OR “psychogenic non-epileptic seizure*” OR “psychogenic non-epileptic seizure*” OR “psychogenic nonepileptic seizure*” OR “non-epileptic seizure*” OR “functional seizure*” OR “dissociative seizure*” OR “psychogenic seizure*” OR “pseudoseizure*” OR “pseudo-seizure*”) AND (“personality disorder*” OR “personality disease*” OR “borderline personality disorder*” OR “cluster-a personality disorder*” OR “cluster-b personality disorder*” OR “cluster-c personality disorder*” OR “narcissistic personality disorder*” OR “avoidant personality disorder*” OR “dependent personality disorder*” OR “obsessive-compulsive personality disorder*” OR “passive-aggressive personality disorder*” OR “schizotypal personality disorder*” OR “schizoid personality disorder*” OR “paranoid personality disorder*” OR “depressive personality disorder*” OR “antisocial personality disorder*” OR “histrionic personality disorder*”).

The * indicates that any combination of letters after the initial string was accepted. All search words were not case-sensitive.

**Figure 1:**
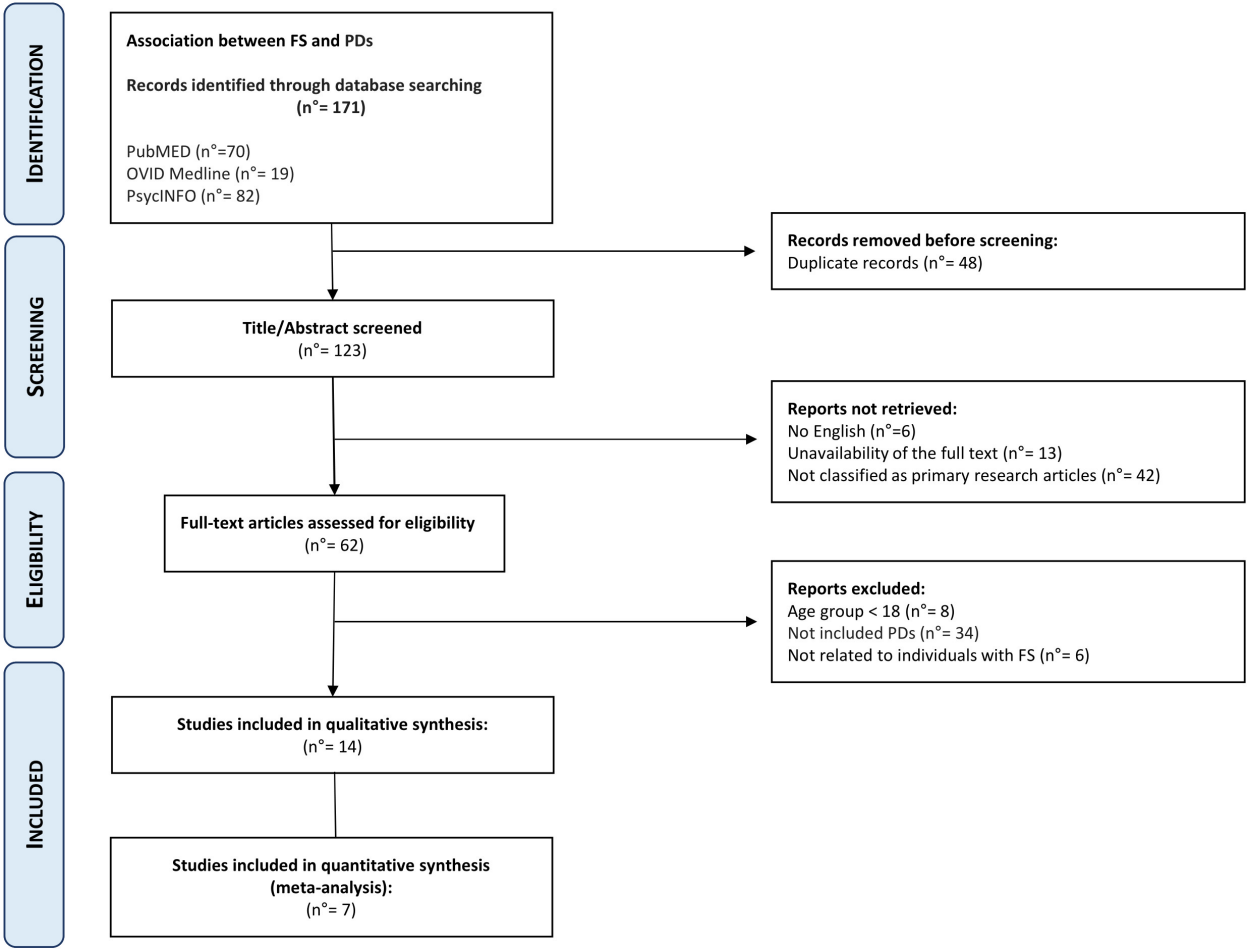
Preferred reporting items for systematic reviews and meta-analyses (PRISMA) study flowchart.

This search, performed on 03 February 2024, returned 70 articles on PubMED, 19 on OVID Medline, and 82 on PsycINFO (**Figure 1**).

After the removal of duplicated articles, the combined searches produced 123 articles. No further papers were identified after searching the reference list of the included articles.

### 2.2 Papers’ assessment for eligibility

Each qualifying article underwent a thorough evaluation based on the following inclusion and exclusion criteria: availability of the full text, written in English, primary research article (case reports with ≥5 individuals were included), related to humans with FS, studies enrolling individuals aged ≥18 years, and PDs diagnosed according to DSM-IV/V or ICD-10/11. Restrictions on the publication’s year were not applied. Papers regarding PDs in infants or adolescents were not considered for this review because our aims included the frequency of all PDs, and the diagnosis of antisocial PD requires at least an age of 18 years in accordance with DSM-V criteria (2).

Three independent reviewers (IM, IS, and FF) assessed each article by title and abstract/full text to determine eligibility, comparing their results. Any discrepancies were resolved through consensus among the review team (IM, IS, FF, LM, and AG).

### 2.3 Data extraction

For each article, the following data were extracted: study setting; study population and participants’ demographics and baseline characteristics; study design; year of publication; main outcome (frequency of PDs in individuals with FS); secondary outcomes (frequency of any PD phenotype in people with FS and, where available, in those with epilepsy); and information for risk of bias assessment. Three people independently extracted the data. Any disagreements were resolved by reaching a consensus within the review team.

### 2.4 Risk of bias

Two sources of bias were considered: inclusion bias and reporting bias in the included studies. To mitigate inclusion bias, each article was assessed by three independent and blinded reviewers. Any discrepancies between reviewers were resolved by reaching a consensus within the three-person review team. After the included articles were selected, the list was assessed by an additional reviewer (AG), and any problematic articles were thoroughly discussed within the team. We also rated each article according to the modified Newcastle-Ottawa Scale (NOS) for cross-sectional studies (22).

### 2.5 Statistical analysis (meta-analysis)

For articles that compared the prevalence of PDs and their relative clusters between FS and epileptic seizure groups, a meta-analysis was conducted using Cochrane’s Review Manager Web tool (RevMan Web) (https://revman.cochrane.org) (23). As the considered variables are dichotomous outcomes, the Mantel-Haenszel method was used to provide the odds ratio (OR) along with its 95% confidence interval (CI) pooled in a forest plot. Heterogeneity across the analyzed studies was evaluated through the Cochrane Q test (χ^2^ test) and the I-squared index (I^2^). A p-value of <0.05 was considered statistically significant. Publication bias was assessed using funnel plots.

## 3 Results

### 3.1 Study selection

As reported in **Figure 1**, out of the 123 articles reviewed, 13 were excluded due to full-text unavailability, 6 articles were not in English, 42 were not classified as primary research articles, 8 articles regarded individuals with age <18 years, 34 had not been diagnosed with PDs according to DSM-IV/V or ICD-10/11, and 6 articles were not related to FS individuals.

After manual assessment, 14 (**Table 1**) articles met the inclusion criteria and were retained for further analysis. The scoring of all reviewed articles is detailed in **Supplementary Table 1**. At the risk of bias assessed through NOS, 4/14 (24–27) and 10/14 (17, 28–36) studies obtained medium-quality (4–6 stars) and high-quality (at least 7 stars) judgments, respectively (**Table 2**).

**Table 1:** Characteristics and results of the included studies.

Author	Year	FS, n°	Age	Diagnostic criteria	Other groups enrolled	Personality assessment	PDs, n° (%)	Cluster A, n° (%)	Cluster B, n° (%)	Cluster C, n° (%)	Other PDs, n° (%)
Bailles et al. (24)	2004	30	34.1 ± 12.7	DSM IV	None	SCID II	18/30 (60%)	1/18 (5.6%)	12/18 (66.7%)	2/18 (11.1%)	3/18 (16.7%)
Binzer et al. (28)	2004	20	27	DSM IV	20 with epilepsy	SCID II	13/20 (65%)	–	7/13 (53.8%)	–	–
D’alessio et al. (29)	2006	24	33.42 ± 14.08	DSM IV	19 with FS + epilepsy	SCID II	17/24 (71%)	1/17 (5.9%)	8/17 (47.1%)	8/17 (47.1%)	–
Direk et al. (30)	2012	35	29.1 ± 9.2	DSM IV	35 with epilepsy 37 healthy subjects	SCID II	26/35 (74%)	1/26 (3.8%)	21/26 (80.7%)	13/26 (37.1%)	–
Harden et al. (31)	2009	16	45	DSM IV	16 with epilepsy	SCID II	13/16 (81%)	4/13 (30.7%)	11/13 (84.6%)	1/13 (7.7%)	–
Hovorka et al. (25)	2007	56	29.6 ± 10,1	ICD-10	None	Structured psychiatric interview	25/56 (44.6%)	1/25 (4%)	18/25 (72%)	4/25 (16%)	2/25 (8%)
Labudda et al. (32)	2018	67	37.7 ± 12.3	ICD-10	42 with FS + epilepsy	SCID II	24/67 (35.8%)	–	10/24 (41.7%)	5/24 (20.8%)	9/24 (37.5%)
LaFrance et al. (26)	2010	38	36.2 ± 13.2	DSM IV	None	SCID II	20/38 (52.6%)	–	10/20 (50%)	–	–
Nezˇa´dal et al. (27)	2011	111	31.2 ± 9.7	DSM IV	None	Psychiatric evaluation	52/111 (46.8%)	1/52 (1.9%)	33/52 (63.5%)	8/52 (15.4%)	10/52 (19.2%)
Rady et al. (33)	2021	33	31.15 ± 7.92	DSM IV	33 with epilepsy	SCID II	29/33 (87.9%)	14/29 (48.3%)	23/29 (79.3%)	22/29 (75.9%)	–
Salinsky et al. (34)	2019	73	46	DSM IV	64 with epilepsy	SCID II	30/73 (41.8%)	–	–	–	–
Scévola et al. (17)	2013	35	37.54 ± 14.07	DSM IV	49 with epilepsy	SCID II	25/35 (71.43%)	5/25 (20%)	15/25 (75%)	11/25 (44%)	–
Stone et al. (35)	2004	20	27	DSM IV	30 with functional disorder different form FS	SCID II	13/20 (65%)	–	7/13 (53.8%)	–	–
Turner et al. (36)	2011	22	40.2 ± 14.5	DSM IV	21 with epilepsy 10 with FS + epilepsy	SCID II	4/22 (18%)	1/4 (25%)	2/4 (50%)	–	1/4 (25%)

DSM, Diagnostic and Statistical Manual of Mental Disorder; FS, functional seizures; ICD, International Classification of Disease; PDs, personality disorders; SCID-II, Structured Clinical Interview for DSM-IV Axis II disorders.

**Table 2:** Quality assessment of included articles through the modified Newcastle-Ottawa Scale for cross-sectional studies.

References	Selection^a^				Comparability^b^	Outcome^c^		Total quality score
	(1) Representativeness of the sample	(2) Sample size	(3) Non-respondents	(4) Ascertainment of exposure	(1) Comparability of individuals based on the design or analysis	(1) Assessment of the outcome	(2) Statistical test	
Bailles et al. (24)	*	*	*	*		**		6
Binzer et al. (28)	*	*	*	*	**	**		8
D’alessio et al. (29)	*	*	*	*	**	**		8
Direk et al. (30)	*	*	*	*	**	**		8
Harden et al. (31)	*	*	*	*	**	**		8
Hovorka et al. (25)	*	*	*	*		**		6
Labudda et al. (32)	*	*	*	*	**	**		8
LaFrance et al. (26)	*	*	*	*		**		6
Nezˇa´dal et al. (27)	*	*	*	*		**		6
Rady et al. (33)	*	*	*	*	**	**		8
Salinsky et al. (34)	*	*	*	*	**	**		8
Scévola et al. (17)	*	*	*	*	**	**		8
Stone et al. (35)	*	*	*	*	**	**		8
Turner et al. (36)	*	*	*	*	**	**		8

a A maximum of 5 stars can be awarded for the selection.

b A maximum of 2 stars can be awarded for comparability.

c A maximum of 3 stars can be awarded for the outcome.

### 3.2 Features of the included studies

**Table 1** summarizes the clinical characteristics of the enrolled individuals, the edition of the DSM or ICD used for PD diagnosis, the methods used for personality assessment, and the main results of all 14 studies included. In all papers included, participants have no intellectual disability, comorbidity, or other relevant conditions, such as epilepsy or drug abuse. Noteworthily, individuals with FS alone were considered for analysis. The sample size varied from 20 to 111 across studies. The age of FS participants ranged from 18 years to 65 years (mean age: 34.9). Concordantly to the literature, the female sex is preponderant across studies, reaching 65% of the study population. In 13/14 studies (17, 24–31, 33–36), the FS diagnosis required the recording of typical events on video-EEG, reaching the “documented” level in accordance with the latest diagnostic criteria (1). In one study (32), FS were determined according to the criteria of the Non-epileptic Seizures Task Force of the International League Against Epilepsy (ILAE), including individuals with diagnostic levels indicated as “probable,” “clinically established,” and “documented” (1). Groups used as FS comparisons were not homogenous among the included studies. In detail, 5/14 studies enrolled people with epilepsy (17, 28, 31, 33, 34), 2/14 individuals with FS plus epilepsy (29, 32), and 1/14 individuals with a diagnosis of a functional disorder different from FS (35); in two cases, individuals with FS and individuals with epilepsy were evaluated in comparison to healthy subjects (30) and people with FS plus epilepsy (36), respectively.

### 3.3 Personality disorders in FS

The rate of PDs as comorbidity in FS ranged from 18% to 87%, with a mean of 53.7%. Diagnosis of PDs was made according to DSM-IV in 12/14 studies (17, 24, 26–31, 33–36) and ICD-10 in 2/14 (25, 32). In total, 12 out of 14 studies used the Structured Clinical Interview for DSM-IV Axis II Disorders (SCID II) to assess PDs (17, 24, 26, 28–36), and in 2/14 cases, diagnosis was based on psychiatric evaluation (25, 27).

Ten out of 14 studies classified PDs in clusters (17, 24, 25, 27, 29–33, 36), and and in 3/14 cases only cluster B has been determined (26, 28, 35). Considering the 13/14 studies that analyzed cluster B, the rate of this personality phenotype varied between 41.7% and 84.6%, with a mean of 63.4% (26, 28, 35). Notably, 10 out of 14 studies assessed the prevalence of cluster A and cluster C. Cluster A subtype ranged from 0% to 48.3%, with a mean of 12.4%. Cluster C demonstrated a frequency between 7.7% and 75.9%, with a mean of 31.6% (17, 24, 25, 27, 29–33, 36). Four out of 14 studies described other PD phenotypes that did not meet the criteria for a specific personality cluster (24, 25, 27, 32). In detail, two studies described 12 individuals with organic PDs (5.1%) (25, 27), one reported three PDs not otherwise specified (1.3%) (24), the remaining counted seven personality changes after stress (3%) and two cases with combined PDs (0.9%) (32).

Moreover, 7 out of 14 studies differentiated subtypes of PDs within each cluster (24, 25, 27, 30, 32, 33, 36). Noteworthy, an individual could receive more than one PD diagnoses. Within cluster A, 11 individuals had paranoid PD (6.2%), 9 had schizoid PD (5.1%), and 7 had schizotypal PD (3.9%). Regarding cluster B, 2 individuals were diagnosed with antisocial PD (1.1%), 101 with BPD (56.7%), 19 with histrionic PD (19%), and 20 with narcissistic PD (11.2%). In relation to cluster C, 35 individuals had avoidant PD (35%), 24 dependent PD (13.5%) and 19 obsessive-compulsive PD (19%). One study reported 16 cases of passive-aggressive PD and 15 cases of depressive PDs, in accordance with the criteria reported in Appendix B of DSM-IV (33). These PDs are no longer listed in the DSM-V but fall under the category of other specified/unspecified personality disorder subclinical diagnoses (2).

**Figure 2:**
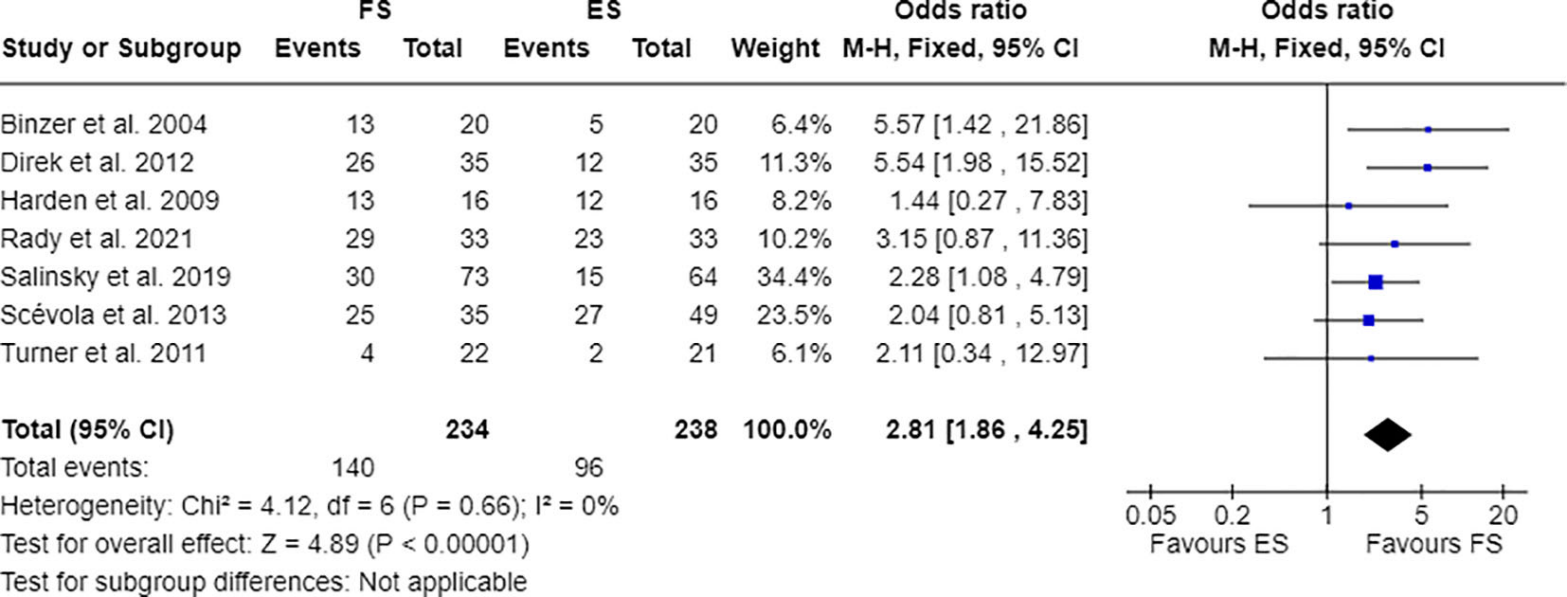
Meta-analysis of eligible studies comparing PDs between FS individuals and the epilepsy population (ES).

**Figure 3:**
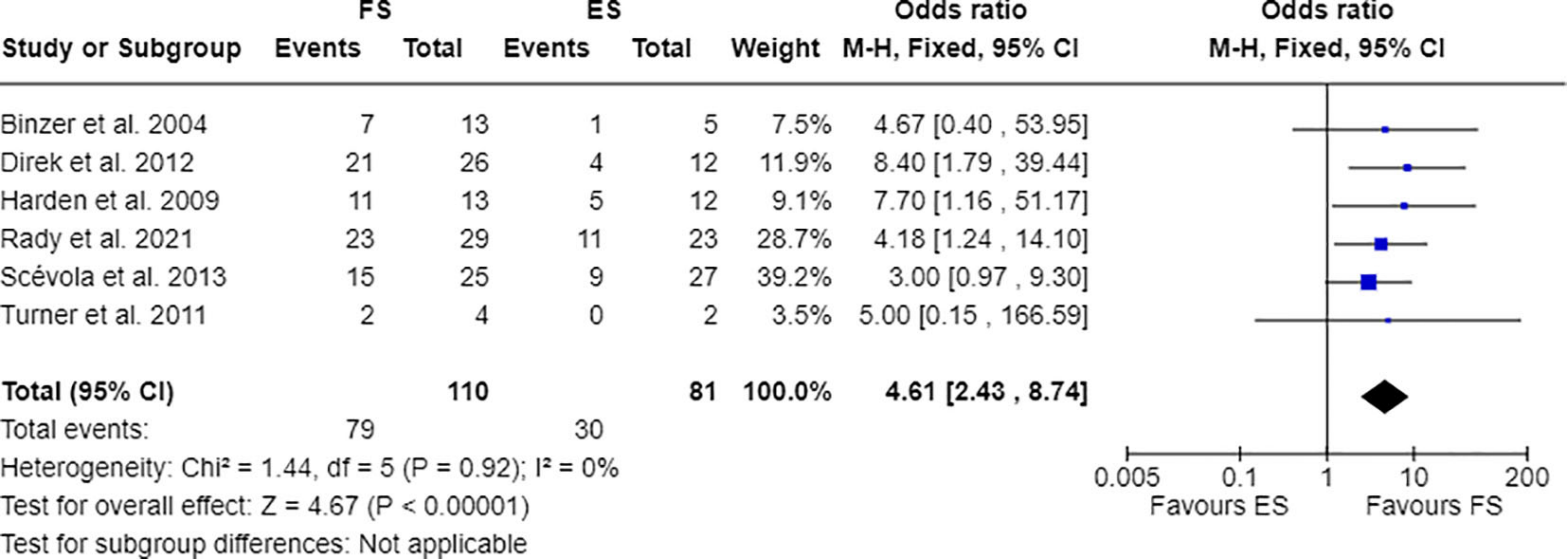
Meta-analysis of eligible studies comparing Cluster B PDs between FS individuals and the epilepsy population (ES).

**Figure 4:**
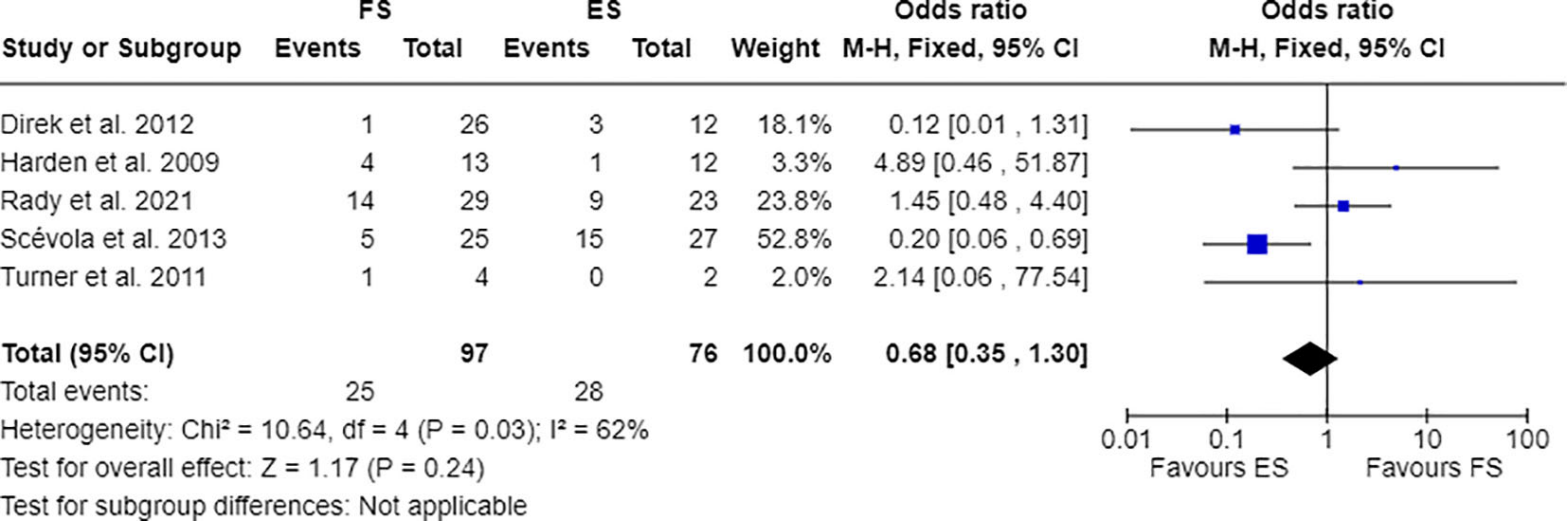
Meta-analysis of eligible studies comparing Cluster A PDs between FS individuals and the epilepsy population (ES).

**Figure 5:**
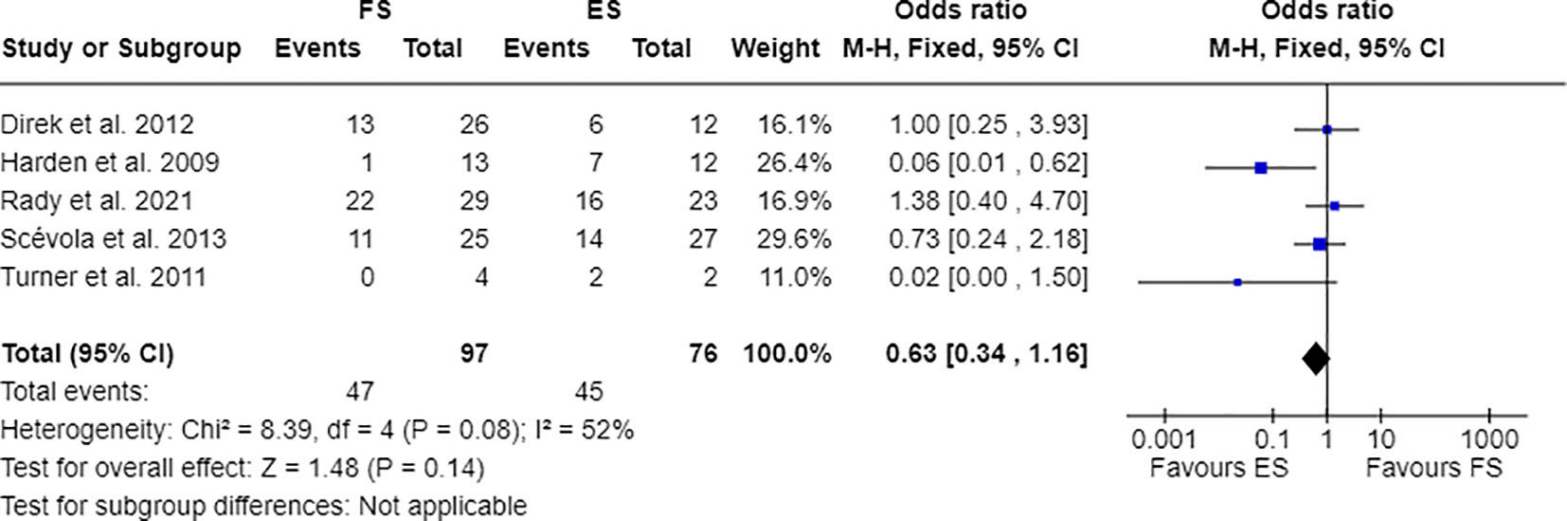
Meta-analysis of eligible studies comparing Cluster C PDs between FS individuals and the epilepsy population (ES).

Furthermore, 7 out of 14 studies used individuals with epilepsy as a comparison group to the FS cohort (17, 28, 30, 31, 33, 34, 36). Combining these studies suitable for meta-analysis, OR resulted greater than 1, indicating that individuals with FS have nearly three times the odds to have PDs than people with epilepsy (OR=2.81; 95% CI=1.86-4.25; I2=0%; p<0.00001), as reported in **Figure 2**. Testing Cluster B prevalence, individuals with FS demonstrated a four-time increased OR to have this PD phenotype compared to epilepsy population (OR= 4.61; 95% CI=2.43-8.74; I2=0%; p<0.00001), as displayed in **Figure 3**. Five out of 14 studies evaluated differences in cluster A and cluster B between FS individuals and people with epilepsy. In detail, people with FS demonstrated an OR less than 1 for cluster A PDs compared to the epilepsy population (OR=0.68; 95% CI=0.35–1.30; I^2 =^ 62%; p=0.024), as represented in **Figure 4**. Similarly, FS individuals proved an OR less than 1 to present a cluster C in comparison with people with epilepsy (OR=0.63; 95% CI=0.34–1.16; I^2 =^ 52%; p=0.014), as delineated in **Figure 5**. The funnel plot displays a symmetric distribution of included studies, indicating no publication bias (**Supplementary Figures 1–4**).

## 4 Discussion

This systematic review illustrates that PDs represent a notable comorbidity in the FS population. When analyzing personality phenotypes, cluster B is the most common PD in FS, in accordance with previous literature data (9, 16). Although at inferior rates, cluster C and, even less, cluster A have been also attested. Among all PD phenotypes, BPD represents the most common diagnosis in FS, followed at an inferior rate by avoidant PD. In comparison to people with epilepsy, our meta-analysis demonstrated in FS individuals an increased risk of having concomitant PD, particularly those belonging to cluster B. At the same time, clusters A and C appear less likely to occur in people with FS than in those with epilepsy.

In the literature, the association between FS and PDs is well recognized (13, 16). A previous systematic review documented a PDs’ rate between 5.4% and 74.3%, while in an earlier work this comorbidity varied from 10% to 86% in FS (16). In our analysis, the prevalence of PDs in FS falls subtly higher, ranging from 18% to 87%. Our slightly greater rate is likely due to mandatory criteria in including exclusively studies diagnosing PD in accordance with DSM-IV/V or ICD-10/11. Nonetheless, a wide disparity in PDs prevalence in FS population has been demonstrated among studies. In a previous review, this difference was attributed to a higher rate of PDs in individuals with a longer duration of FS (16). We did not confirm this trend in our work. It is not known whether differences in FS clinical semiology (such as motor and non-motor manifestations) or levels of dissociation could explain this disparity. Since not all studies included in the present review assessed these features, we were unable to conduct such an analysis. Cluster B PDs, particularly BPD, have been widely demonstrated as the most prevalent personality phenotype in FS (16). Our analysis confirmed BPD as the most frequent PD in FS, regarding more than half of this population. Other cluster B PDs have a lower frequency. In particular, although histrionic PD shares some traits with BPD, its frequency appears lower than that expected in FS, with a prevalence not exceeding 20%. Some reports describe cluster C subtypes of personality in FS. According to our data, cluster C PDs, despite their prevalence can reach up to 75.9% in FS, proved a greater association to epilepsy. Similarly, a previous study reported cluster C more frequently in people with epilepsy than in those with FS (37).

BPD combines marked impulsivity, interpersonal relationships, self-image, and affects instability, consisting of rapidly shifts between extremely positive idealization and extremely negative devaluation about self and others (2, 20). The hallmark of BPD is emotional dysregulation, reflecting an inability to respond to and manage emotional transitions (16, 38). Notably, people with FS frequently exhibit emotional dysregulation and instability in interpersonal relationships (9, 16, 18). Additionally, individuals with BPD may commonly experience childhood sexual/emotional abuse or neglect and have higher rates of concurrent psychiatric disorders, such as depression or PTSD (20). Moreover, dissociative symptoms are included among the diagnostic criteria for BPD and may represent a psychological mechanism underlying FS development (7, 20). Likewise, the similarity between these conditions also includes anger problems, hostile coping styles and somatoform disorder in comorbidity. In this context, BPD might be interpreted as a predisposing etiological factor for FS (30). Interpreting psychiatric disorders in FS as mere comorbidities could be misleading (12). The high heterogeneity in FS clinical and psychiatric manifestations likely reflects the wide range of underlying psychopathologies (30). Conversely, psychiatric comorbidities might contribute to poor outcomes in FS, acting as perpetuating factors (6).

Regardless of their relationship, the identification of psychiatric disorders in FS, especially PDs, significantly impacts treatment choice. Currently, psychotherapy is the first-line therapy for both FS and BPD (19, 20). CBT has been widely demonstrated to reduce attack frequency and improve quality of life in individuals with FS (19, 26, 39). To date, two CBT approaches for FS have been evaluated through randomized controlled trials. The CBT developed by Goldstein targets factors involved in the development and maintenance of attacks, interpreting FS as dissociative responses. The CBT model reported by LaFrance promotes behavior and self-control, addressing both seizures and comorbidities (19). Among the psychotherapies available for BPD, DBT was developed in accordance with Linehan’s biosocial theory, which conceptualizes BPD as a pervasive dysregulation disturbance with great emotional vulnerability and a deficient ability to modulate emotions (40). DBT includes skill modules such as mindfulness, interpersonal effectiveness, emotion regulation, and distress tolerance (20, 41). In this regard, mindfulness-based therapy has shown efficacy in FS. As mentioned above, individuals with FS could be overwhelmed by their emotions, becoming detached from them as an adaptive strategy. Mindfulness increases awareness of feelings and reinforces attention to body symptoms and their misattribution (42). In this light, characterizing concomitant psychiatric disorders allows for the individualization of treatment for FS. As mentioned above, individuals with FS may exhibit various psychopathologies, such as a dissociative response to a previous traumatic life event, a maladaptive response to overwhelming situations, or the automatic activation of learned mental representations. As expected, different etiopathologies and underlying defense mechanisms have been demonstrated to influence treatment. However, psychotherapy for FS tailored to concomitant psychiatric disorders has not yet been thoroughly investigated.

The present review has some limitations. The main limitation is the small FS sample size in each included study. Nevertheless, FS diagnosis appears quite homogeneous across studies, reaching the “documented” level in almost all cases (1). Moreover, half of the included studies evaluated the prevalence of each PD subtype, further restricting the data analysis. Additionally, the comparison groups to FS are sufficiently heterogeneous, comprising healthy subjects, people with epilepsy, and individuals with FS plus epilepsy. The epilepsy population was the most numerically represented across the included studies, allowing for a meta-analysis.

In conclusion, PDs have been shown to be a common comorbidity in FS. Cluster B, especially BPD, demonstrated a high prevalence in individuals with FS. Evaluating the presence of PDs, particularly BPD, may have relevance in personalizing FS treatment. Psychotherapy tailored to concomitant psychiatric disorders could be more effective in reducing the recurrence of attacks and improving quality of life. Systematic investigation of therapeutic approaches structured around psychiatric comorbidity is currently not available (39). Further studies are needed to clarify the potential benefits of selecting psychotherapy based on the psychiatric comorbidity of an individual, especially PDs.

## Data availability statement

The original contributions presented in the study are included in the article/**Supplementary Material**. Further inquiries can be directed to the corresponding author.

## Author contributions

IS: Conceptualization, Data curation, Formal analysis, Writing – original draft. IM: Conceptualization, Data curation, Writing – review & editing. LM: Conceptualization, Data curation, Writing – review & editing. FF: Conceptualization, Formal analysis, Methodology, Writing – review & editing. AG: Conceptualization, Supervision, Writing – review & editing.
