# Supplementary material for: Personality disorders in individuals with functional seizures: a systematic review
Source: Front Psychiatry. 2024 Aug 1;15:1411189. doi: 10.3389/fpsyt.2024.1411189 (PMC11333798; doi:10.3389/fpsyt.2024.1411189)

Supplementary Material

Personality disorders in individuals with Functional Seizures: a systematic review

Ilaria Sammarra†^1^, Iolanda Martino†^1^, Laura Marino^1^, Francesco Fortunato^1^, Antonio Gambardella^*1^

^1^Institute of Neurology, Department of Medical and Surgical Sciences, Magna Graecia University of Catanzaro, Italy

† These authors share first authorship

*** Correspondence:** Prof. Antonio Gambardella
a.gambardella@unicz.it

## Supplementary Figures


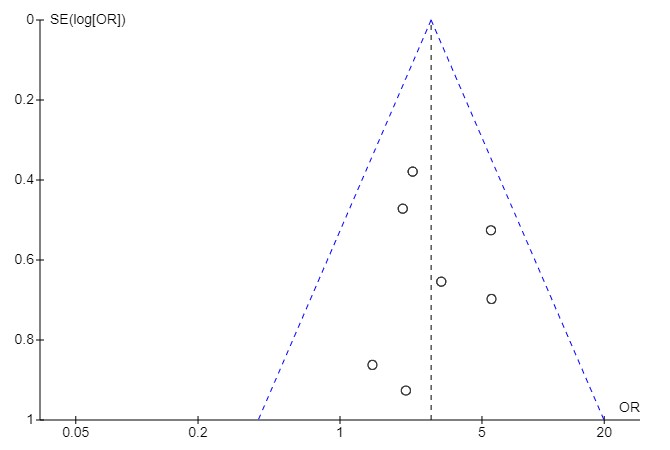


**Supplementary Figure 1** Funnel plot of PDs between FS individuals and epilepsy population (ES).


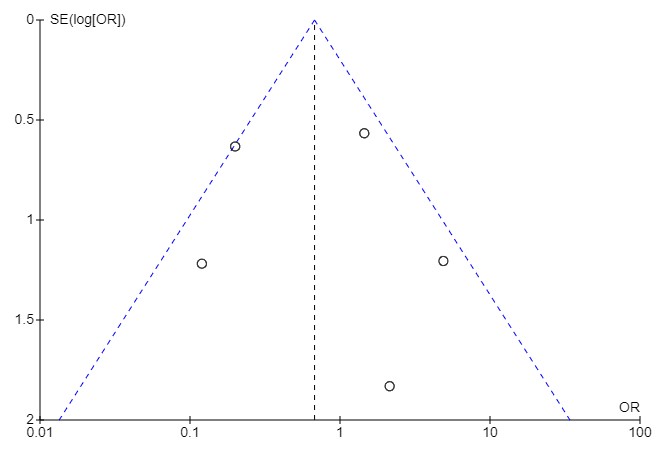


**Supplementary Figure 2** Funnel plot of Cluster B PDs between FS individuals and epilepsy population (ES).


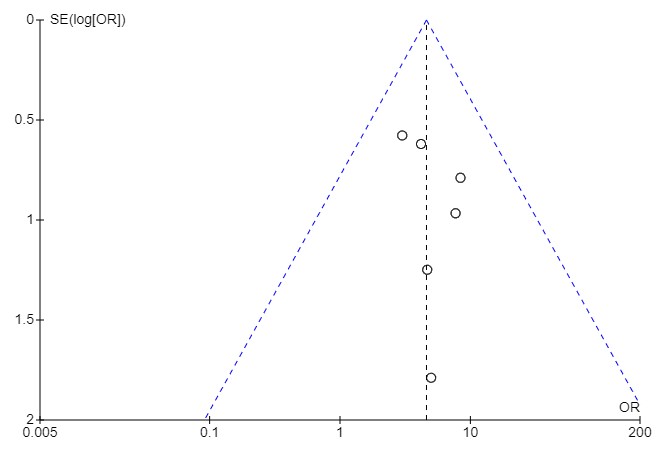


**Supplementary Figure 3** Funnel plot of Cluster A PDs between FS individuals and epilepsy population (ES).


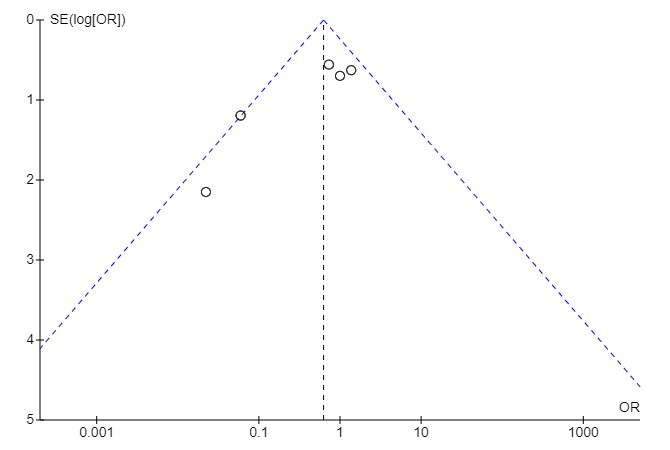


**Supplementary Figure 4** Funnel plot of Cluster C PDs between FS individuals and epilepsy population (ES).

**Supplementary Tables**

**Supplementary Table 1** Scoring of all reviewed articles obtained from PubMED, OVID Medline and PsycINFO.

**Supplementary Data** PRISMA (Preferred Reporting Items for Systematic Reviews and Meta-Analyses) checklist.


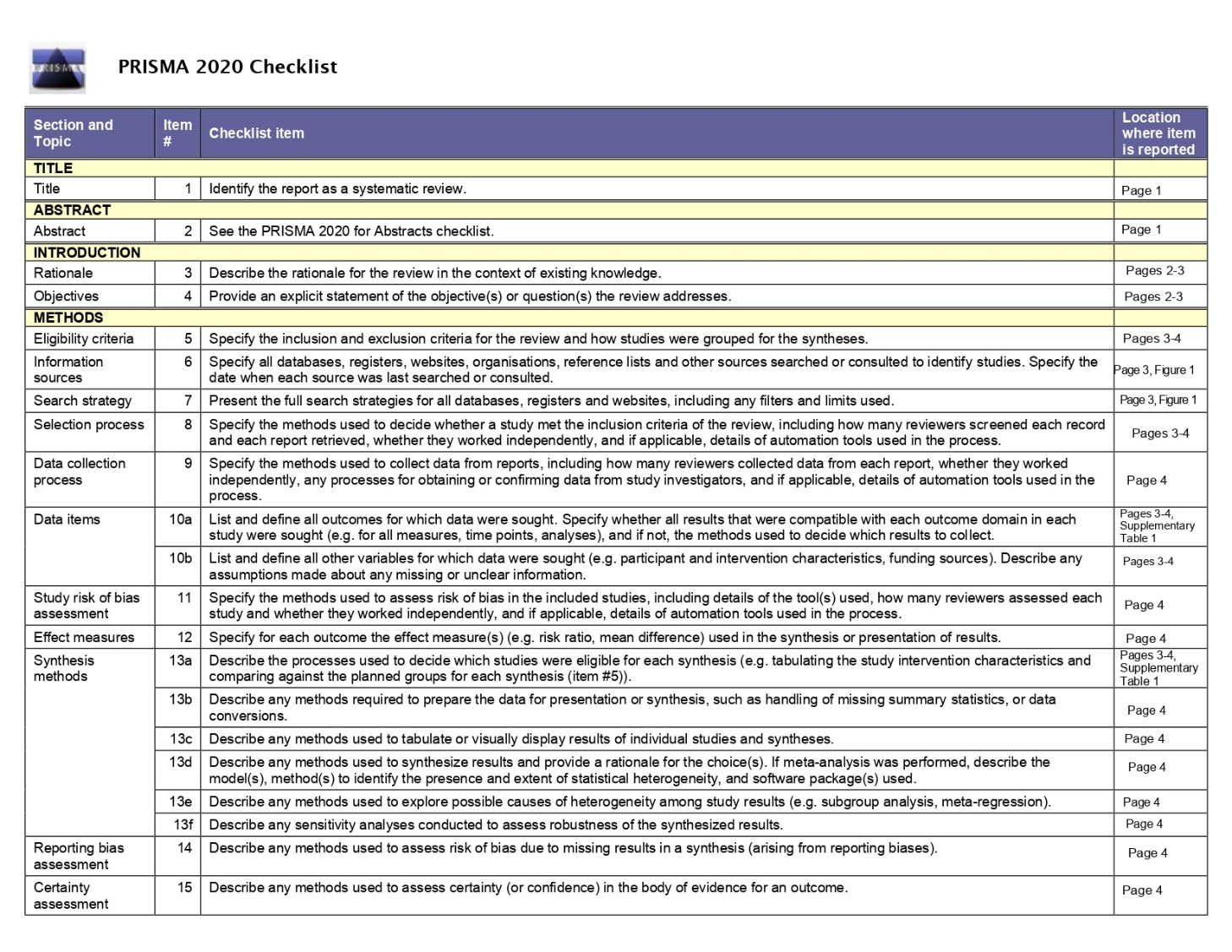


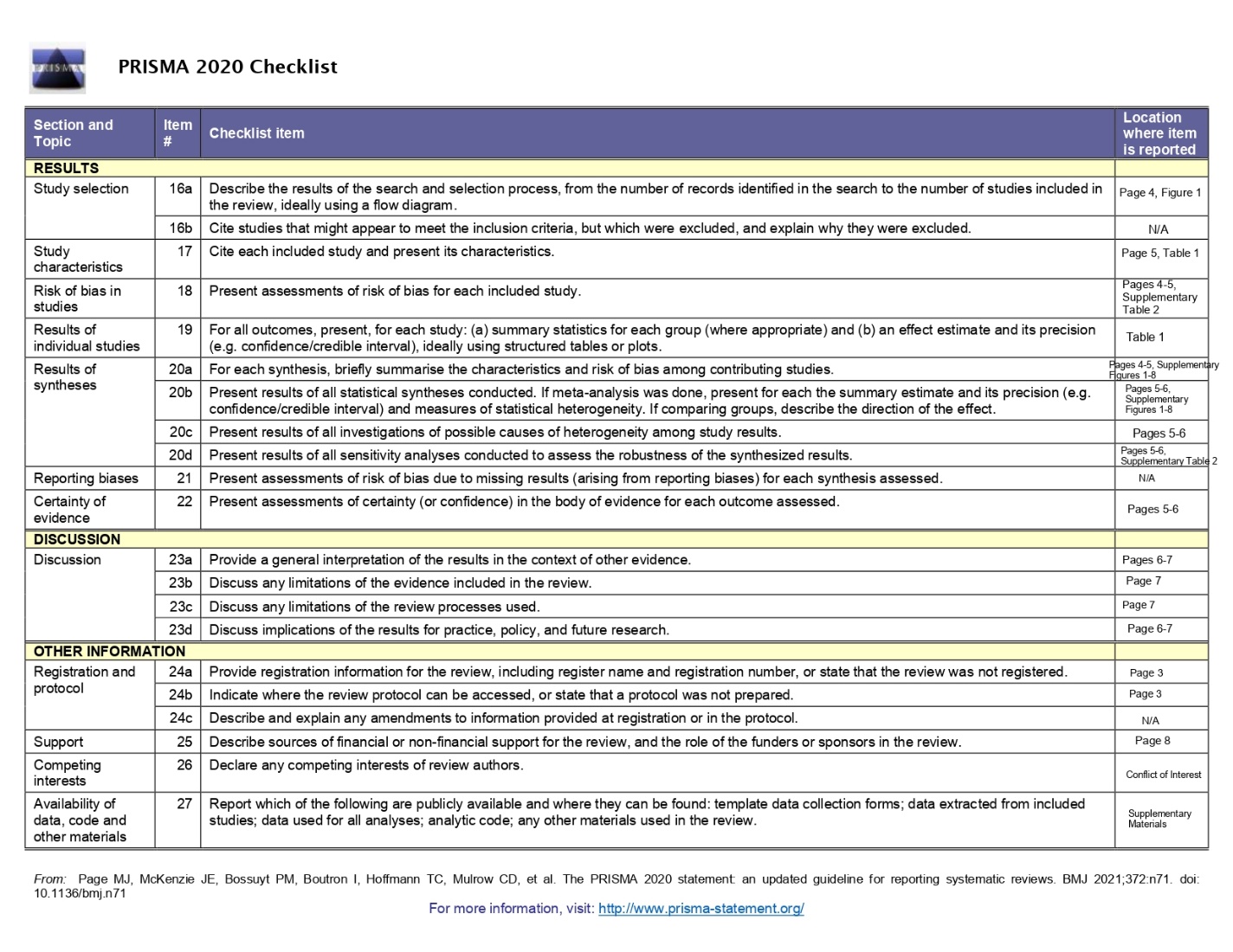

Supplement: Supplementary file 1 [file DataSheet_1.docx]
